# The promise of data science for health research in Africa

**DOI:** 10.1038/s41467-023-41809-2

**Published:** 2023-09-29

**Authors:** Clement A. Adebamowo, Shawneequa Callier, Simisola Akintola, Oluchi Maduka, Ayodele Jegede, Christopher Arima, Temidayo Ogundiran, Sally N. Adebamowo, BridgELSI Project as part of the DS-I Africa Consortium

**Affiliations:** 1 Department of Epidemiology and Public Health, and Greenebaum Comprehensive Cancer Center, University of Maryland School of Medicine, Baltimore, MD USA; 2 Department of Research, Center for Bioethics and Research, Ibadan, Nigeria; 3 Department of Clinical Research and Leadership, School of Medicine and Health Sciences, The George Washington University, Washington DC, USA; 4 Center for Research on Genomics and Global Health, National Human Genome Research Institute, National Institutes of Health, Bethesda, MD USA; 5 Department of Business Law, Faculty of Law, University of Ibadan, Ibadan, Nigeria; 6 Department of Bioethics and Medical Humanities, Faculty of Multidisciplinary Studies, University of Ibadan, Ibadan, Nigeria; 7 Department of Sociology, University of Ibadan, Ibadan, Nigeria; 8 Syracuse University College of Law, Syracuse, NY USA; 9 Department of Surgery, College of Medicine, University of Ibadan, Ibadan, Nigeria

**Keywords:** Scientific data, Data processing, Health policy, Ethics, Decision making

## Abstract

Data science health research promises tremendous benefits for African populations, but its implementation is fraught with substantial ethical governance risks that could thwart the delivery of these anticipated benefits. We discuss emerging efforts to build ethical governance frameworks for data science health research in Africa and the opportunities to advance these through investments by African governments and institutions, international funding organizations and collaborations for research and capacity development.

## Abstract (web summary)

In this Review article, the authors discuss emerging efforts to build ethical governance frameworks for data science health research in Africa and the opportunities to advance these through investments by African governments and institutions, international funding organizations and collaborations for research and capacity development.

## Introduction

Data science is poised to revolutionize healthcare and research by enabling the development of novel interventions and groundbreaking strategies derived from high-quality and efficient analyses of the huge datasets derived from the activities of our multifaceted lives. Data science applications use high-performance computational infrastructure to process massive datasets from personal, public, and commercial sources including healthcare systems, smartphones, shopping records, social media postings, and wearable devices. Using novel, complex, and occasionally opaque algorithms, data scientists generate new insights and generalizable knowledge. Examples of data science applications include combination of diverse data streams to develop bio-preparedness, monitoring, and response strategies for infectious diseases outbreaks in human health and in agriculture^1^. Other examples include the use of Geographical Information System (GIS) data to map spatial variations in the determinants, incidence, prevalence, and outcomes of disease, and the response of healthcare systems^2,3^. Several countries also utilize data science to monitor and evaluate multi-sectoral progress towards meeting the UN Sustainable Development Goals (SDG), reduce fraud and corruption, identify fake pharmaceutical products, and improve supply chain management to prevent stock-outs^4–11^. More recently, Large Language Models (LLM) have captured public imagination with the release of tools like GPT-4. LLMs are rapidly being used to transform multiple industries and disciplines^12–17^. With these capabilities, data science is rapidly transforming the landscape and touching vast areas of human endeavors.

Data science health research is the novel application of data science methods and technologies for systematic generation, collection, processing, storage, management, analyses, visualization, interpretation, and communication of health-related data to develop generalizable knowledge and generate actionable insights. Examples of data science health research include integration of data from multiple omics technologies to generate insights about biological mechanisms and diseases’ pathways, and identify novel therapeutic and preventive opportunities^18^. Other examples include mining electronic health records for precision medicine^19^ and analysis of medical images for computational histopathology and radiology to improve diagnoses, among other uses^20,21^. In public health, data scientists are transforming practice through the application of high-level computational methods to population-level datasets typically used in public health to advance precision public health practice and research^22^. Examples of public health data science research include mining social media data for early detection, analyses of trends, education, planning and implementation of health systems responses to infectious and non-communicable diseases^8,9,11,23^, and application of myriad novel technologies and algorithms for precision public health^22^. Data science health research therefore presents huge opportunities for the application of novel methods and transformative technologies that would solve many healthcare challenges facing African people today and enable wider availability of high quality and cost-efficient health services.

Africa, potentially, has the most to gain from implementation of data science for health care and research. With a population expected to reach 2.5 billion people or ~25% of the world’s population by 2050, data science technologies would enable African countries to leapfrog legacy healthcare systems and technologies, and dramatically transform lives on the continent^24,25^. Even though Africa currently constitutes 17% of the world’s population, it bears 25% of the world’s disease burden, has only 3% of the world’s healthcare workers, and 2% of global health research output^26^. This is due to limited infrastructure, lack of trained personnel, poor funding, economic and social instability which hinder access to clinical and preventive services^26^. Global public health emergencies such as emerging and re-emerging infectious diseases epidemics and climate change pose more challenges to African countries than the rest of the world. African countries would therefore require innovative data science tools and strategies to overcome these challenges.

Substantial gaps exist in the representation of people from Africa in the datasets currently used to build data science models and applications^27^. This underrepresentation renders data science models and algorithms unstable and potentially inaccurate in African populations^27^. Without dedicated and focused efforts at remediation, persistence of this data science equity gap would worsen and this portends dire consequences for data science health research in African populations.

African researchers, institutions, governments, and the private sector are already using data science for research, discoveries, and preventive and clinical care^28,29^. Most of these uses involve novel applications or extensions of current healthcare expertise and technologies. Examples of data science applications already in use in Africa include teleradiology and telepathology, patients’ navigation and clinical decision support (CDS) tools, integration of genomics data into public health and clinical care, and cancer screening^29–33^. However, most of these applications were designed, developed, tested, and validated outside Africa. They may not have been adequately evaluated in African populations and may be insensitive to local contexts and health priorities^28,34,35^. They may therefore primarily benefit people outside the continent. Given the novel opportunities being created by data science applications, it is critical to develop and implement technologies that are relevant and adapted to the contexts in which they would be used in Africa.

## Investment in data science health research infrastructure in Africa

Several initiatives are being implemented in Africa to develop data science health research capacity, build infrastructure, implement training programs, organize scientific conferences, and engage in international collaborations that would empower African institutions to generate datasets, develop and apply data science models, and close the data science gap between Africa and high-income countries (HIC). In 2022, the NIH Common Fund awarded 20 grants worth $74.5 million in the “Harnessing Data Science for Health Discovery and Innovation in Africa (DS-I Africa)” program to accelerate data science health research in Africa. The projects being implemented by the DS-I Africa program include a Coordinating Center, an Open Data Science platform, seven training programs, four ethical, legal, and social implications (ELSI) projects, and seven research projects. (Table 1)^36^.

**Table 1:** Harnessing data science for health discovery and innovation in Africa (DS-I Africa) projects, collaborating institutions and study aims

No.	Project title	Grant type	Collaborating institutions	Objectives
1.	Advancing Public Health Research in Eastern Africa through Data Science Training (APHREA-DST)	Training program	Columbia University, USA Addis Ababa University, Ethiopia University of Nairobi, Kenya	Develop new context-specific MS in public health data science. Mentor faculty to build and strengthen capacity in health data science. Conduct short-term training programs.
2.	Computational Omics and Biomedical Informatics Program (COBIP)	Training program	Oregon Health & Science University (OHSU), USA University of Cape Town, South Africa.	Develop data science training program focused on the health priorities of Africa. Implement multi-disciplinary in biomedical data science. Establish an international center of excellence in computational omics and biomedical informatics in Africa.
3.	Data Science for Child Health Now in Ghana (DS-CHANGE)	Training program	KNUST, Ghana University of Washington, Seattle Children’s Hospital and Research Institute Smile Train	Deliver a comprehensive mentored interdisciplinary training program in data science health research. Increase capacity in biomedical data science. Develop proficiency in effective team science methods for interdisciplinary teams.
4.	Makerere University Data Science Research Training to Strengthen Evidence-Based Health Innovation, Intervention and Policy (MakDARTA)	Training program	Makerere University, Uganda Johns Hopkins University, USA	Establish graduate degree training in data science at MakCHS. Provide Postdoctoral training. Train MakCHS faculty to enhance their capabilities for data science research and mentorship of data science trainees.
5.	NYU-Moi Data Science for Social Determinants Training Program (DSSD)	Training program	New York University, USA Moi University, Kenya Brown University, USA	Create intellectual meeting spaces for a data science and health. Propel and sustainably advance data science capacity in Kenyan institutions.
6.	Research Training on Harnessing Data Science for Global Health Priorities in Africa	Training program	Harvard University, USA Heidelberg University, Germany University of KwaZulu-Natal, South Africa	Train mid-level and senior researchers at UKZN. Build a critical mass of junior public health and medical professionals across South Africa, Ghana, Nigeria, Tanzania, and Uganda. Implement sustainable master’s degree program in health data science at the UKZN.
7.	Research Training in Data Science for Health in Rwanda	Training program	Washington University in St. Louis, USA University of Rwanda, Rwanda African Institute for Mathematical Sciences, Rwanda	Identify competencies in major scientific areas. Foster innovative team science transdisciplinary approach to research. Build institutional capacity to support long- term sustainability.
8.	Combatting Antimicrobial Resistance (AMR) in Africa Using Data Science (CAMRA)	Collaborative research center	Redeemer’s University, Nigeria J. Craig Venter Institute, Inc., USA University of Nebraska Medical Center, USA	Comparative phenotypic and genotypic studies of clinical isolates to inform trends in AMR and dynamics of transmission. Develop portable screening tool for clinical care. Explore benefit of an aminoglycoside conjugated to an antimicrobial peptide for enhanced bactericidal activity.
9.	Developing data science solutions to mitigate the health impacts of climate change in Africa: the HE2AT Center	Collaborative research center	University of the Witwatersrand, South Africa Liverpool School of Tropical Medicine (LSTM), UK IBM Research Africa, South Africa University of Basel, Switzerland University of Washington, USA	Prepare a data science ecosystem and platform to support analysis of existing environmental, biomedical, and other data. Increase knowledge and develop sustainable solutions to address climate change and health. Advance capacity for data science research in climate change and health in sub-Saharan Africa. Serve as a continental resource and center of excellence for policy and practice.
10.	Harnessing Data Science to Promote Equity in Injury and Surgery for Africa (D-SINE)	Collaborative research center	University of Buea, Cameron University of California, Los Angeles and Berkeley, USA The Cameroonian Ministry of Public Health African Institute for Mathematical Sciences, Cameroon University of Cape Town, South Africa.	Decrease the burden of injuries and surgical diseases through improved surveillance, prevention, and treatment. Improve access to quality surgical care in Cameroon and other SSA countries.
11.	Multimorbidity in Africa: Digital innovation, visualization, and application (MADIVA)	Collaborative research center	University of the Witwatersrand, South Africa African Population and Health Research Center, Kenya IBM Research Africa South African Population Research Infrastructure Network Vanderbilt University Medical Center. South Africa	Link integrated data sets and create dashboard that will allow researchers to find data and plan projects. Create a clinic dashboard to help clinic managers and health officials monitor and plan effectively. Develop robust methods for risk profiling of individuals and communities using heterogeneous data and pioneer use of polygenic risk scores in Africa.
12.	MUST Data Science Research Hub (MUDSReH)	Collaborative research center	College of Ophthalmologists of Eastern, Central and Southern Africa Kwame Nkrumah University of Science and Technology, Ghana Massachusetts Institute of Technology, USA Massachusetts General Hospital, USA	Use data science to analyze medical images for advancing clinical care. Integrate data science with implementation science to promote clinical impact.
13.	Role of Data Streams In Informing Infection Dynamics in Africa- INFORM Africa	Collaborative research center	Institute of Human Virology Nigeria Centre for the AIDS Programme of Research In South Africa	Establish data streams from public and private sectors to understand multilayer interactions that explain the dynamics and impact of COVID- 19 pandemic. Develop geospatial tools for pandemic surveillance and response to improve preparedness. Expand data science research opportunities and capacity. Maintain sustained engagement with the policy makers and governments.
14.	UtiliZing health Information for Meaningful impact in East Africa through Data Science (UZIMA-DS)	Collaborative research center	Aga Khan University, Kenya University of Michigan, USA	Harmonization of multimodal data sources for meaningful use and analyses. Leveraging temporal patterns of data to identify trajectories through prediction modeling using AI/ML-based methods. Engaging with key stakeholders to identify pathways for dissemination and sustainability of these models into target communities.
15.	Bridging Gaps in the ELSI of Data Science Health Research in Nigeria (BridgELSI)	ELSI project	Center for Bioethics and Research (CBR), Nigeria George Washington University, USA University of Maryland School of Medicine, USA	Evaluate current legal frameworks for ethical oversight of data science health research. Develop new and innovative governance frameworks to support data science health research in Nigeria. Prospectively evaluate the knowledge, attitude, and practices to current and emerging ELSI of data science research in Nigeria.
16.	DS-I Africa - Law	ELSI project	University of Kwazulu-Natal, South Africa	Investigate the legal themes associated with modes of informed consent to the use of data; the nature and content of individual and community rights in genomic data; the use of individuals’ geospatial data for public health surveillance; the cross-border sharing of data; and the use of data as basis for Artificial Intelligence (AI).
17.	Public Understanding of Big data in Genomics Medicine in Africa (PUBGEM-Africa)	ELSI project	University of Cape Town, South Africa	Investigate models of public engagement and preparedness for big data use in health. Explore the roles and responsibilities of stakeholders regarding intellectual property and commercial use of genomics big data in health. Investigate public perceptions of governance of big data in health. Develop data governance frameworks for innovation and health in Africa.
18.	Research for Ethical Data Science in Southern Africa (REDSSA)	ELSI project	Stellenbosch University, South Africa University of North Carolina, USA	Obtain key stakeholder views regarding the governance of data science in DSI-Africa Research Hubs in Southern Africa. Develop guidance documents. Create ELSI networks and communication channels on data science in Southern Africa.
19.	DS-I Africa Coordinating Center	Coordinating Center	University of Cape Town, South Africa	Develop an efficient CC addressing all the joint administrative, collaborative, and logistical needs of the consortium.
20.	Open Data Science Platform	Open Data Science Platform	University of Cape Town, South Africa	Establish the ODSP gateway and its eLwazi platform to facilitate novel discoveries for health.

The DS-I Africa initiative builds on the infrastructure previously developed by programs such as the $176 million Wellcome Trust and NIH-funded Human Heredity & Health in Africa (H3Africa) program^37^. H3Africa built new collaborations among scientists, developed genomics research infrastructure, and created publicly available governance and ethics policies for the African genomics research ecosystem^38–40^. Data science conferences and training programs are also proliferating in Africa including the Data Science Africa—an AI and Data Science Research Group at Makerere University, Uganda, the multi-country African ML and AI organization— Deep Learning Indaba, the School for Data Science and Computational Thinking at Stellenbosch University in South Africa, the African Institute for Mathematical Sciences Centre of Excellence in Cameroon and the African Center of Excellence in Data Science in the University of Rwanda. These programs are critical to generating data that will close the data science gap in Africa and enrich global resources for data science health research.

## Developing a comprehensive framework for the governance of data science health research across Africa

In contrast with other disciplines where data science is also rapidly advancing, health research already has established frameworks and infrastructure for ethical governance. Substantial investments by the US National Institutes of Health (NIH), UK Wellcome Trust, the European Union through the European-Developing Countries Clinical Trials Partnership (EDCTP), African governments and institutions have significantly expanded African health research ethics infrastructure in the past few decades^41–45^. Despite these investments, there remain many unresolved challenges including concerns about quality of informed consent, data ownership, data sharing, benefit-sharing, privacy, autonomy, exploitation, and weak governance^46–49^. Recent examples of these challenges include controversies about community benefit and data sharing during COVID-19 pandemic research and unauthorized use of DNA samples from African populations to develop a DNA genotyping microarray chip^38,50^. Given the methods and technologies used in data science health research, its potential to exacerbate preexisting health research ethics problems and generate new ones are quite substantial.

Research consortia like H3Africa developed policies on samples and data sharing, biorepositories, publications, collaborations, and commercialization^38^. They also provided training for researchers and ethics committees. For example, H3Africa’s publication policy gives African researchers protected time to analyze and publish before their data becomes publicly available. This protection, which is designed to accommodate the infrastructural and personnel challenges faced by African researchers, enables them to frame the narrative about their research and advance their research priorities. Other policies require that the funded studies should focus on African health priorities, be led by African researchers, and that African institutions should be the primary recipients of the research grants, even when they collaborate with international institutions. These are meaningful practices that the emerging data science health research programs should emulate and further develop.

The rapid evolution of data science methods, utilization of complex algorithms, and huge datasets obtained from a variety of sources under uncertain consenting procedures particularly challenges the current model of ethical review of health research^51,52^. When data science health research projects are conducted within single institutions, e.g., computational histopathology of diagnostic biopsies, sufficient ethical oversight can be provided by the institution’s health research ethics committee. However, even in these situations, institutional health research ethics committees may lack sufficient knowledge, expertise, and experience in evaluating the ethical dilemmas that may accompany these studies and struggle to provide adequate ethical review and oversight^53^. In addition, the methods and technologies of data science often run counter to the established principles and practices of ethics review of health research thereby creating situations that may be beyond the capacity of individual ethics committees to resolve^54–56^. In such cases and in others where data science health research is being conducted in multiple institutions within the same country, collaborations between the ethics committees or centralization of ethics review, for example, by national health research ethics committees may be required to provide ethical oversight^57^. National health research ethics committees can constitute standing review committees that, in addition to chartered members, may also include local and international experts as ad-hoc members who can provide ethical oversight for complex data science health research within national boundaries^57^. This centralization of ethical review enables efficient utilization of scarce data science health research ethics expertise and improves the efficiency of ethical review^57,58^. Despite these innovations, even national health research ethics committees are susceptible to some of the problems affecting institutional health research ethics committees including lack of resources, lack of independence, poor funding and lack of efficacy, albeit to a lesser degree^44,45,53^.

## What should be next on the agenda for data science health research ethics in Africa?

### Improve institutional and national health research ethics governance infrastructure

Despite the tremendous investments in recent decades, the capacity, resources, and infrastructure for ethical oversight of health research in Africa remains weak and poorly resourced. A surge of data science health research projects would significantly strain and may overwhelm the system. The major responsibility for building and maintaining national and institutional health research ethics infrastructure rests with African governments and local institutions. Information showing how research significantly boosts the intellectual and economic capital of institutions and countries, and are engines for growing local and national economies may encourage more investment in research infrastructure, including ethical review in Africa. Many African institutions built their current health research ethics programs to support local investigators involved in collaborative international research projects. Research sponsors should incentivize development of local data science health research ethics capacity by linking progress in this domain with new research funding^59^. This would be highly impactful and motivate significant institutional response.

Research ethicists should engage with their local research and data science communities to better understand data science health research methods and projects, and jointly develop ethical governance frameworks that build on existing research ethics oversight infrastructure. Well-funded, well-designed, and sufficiently long training programs that have enabled African countries to avoid widespread egregious harm to research participants despite recent growth in the volume and sophistication of health research on the continent, are also needed for data science health research ethics^42^. These would improve the knowledge of research ethicists about data science health research and that of data scientists about health research ethics, build local capacity that would enable local ownership and sustainment of training programs, and support the conduct of research into contextual data science health research ethics in Africa while contributing to the global health research ethics discourse.

### Develop culturally and resource-level appropriate national laws, guidelines, and regulations, and the infrastructure for enforcement

Many African governments are rushing to enact laws similar to the European Union’s General Data Protection Regulation (GDPR) and modifying them for their environments^60,61^. The major challenges with GDPR and similar data protection laws include lack of sufficiently explicit frameworks for enforcement, complexity of certain provisions, a focus that is often insufficient for the nuances of data science health research^62,63^. Other suggestions for ethical governance of data science include giving participants ownership of their digital selves or using blockchain technologies to protect digital privacy and securely share data^64,65^. These approaches are highly technological, expensive and are not resource-level appropriate in the African health research setting^66^.

Research and training consortia are developing novel policies, ideas, and implementation strategies for ethical regulation of data science health research^67,68^. These consortia must engage frontline stakeholders in different research environments and serve as petri-dishes for experiments into ethical regulation of data science health research. Governments and their agencies also have major roles to play in engendering and maintaining public trust, accountability, and support that are required to sustain public engagement in and support for data science health research.

Existing health research ethics regulations already have the essential elements for the ethical oversight of all types of health research. While data science health research includes novel methods and technologies, these do not abrogate the foundations, principles, and practices of modern health research ethics. African countries can quickly introduce sufficient oversight of data science health research by adding to or modifying existing regulations.

### Develop and implement enforceable multinational regulations

Most data science health research sponsors, principal investigators, and scientists belong to international or commercial organizations that may not have local offices in Africa and may not be subject to national laws, guidelines, and regulations^58^. This poses significant problems for oversight and accountability. Multilateral agencies including the United Nations and its organs, governments, advocates, bioethicists, and researchers have conducted multiple consultations and stakeholders’ meetings leading to issuance of guidelines on the use of data science in healthcare, research, and policy^69,70^. These guidelines call for development of multinational frameworks for data science health research to prevent egregious harm and maximize data science’s benefits to global populations^52^. To ensure relevance and implementation, these multinational agencies should work with African institutions to develop the mechanisms for enforcement of these principles, model laws, guidelines, and regulations for ethical oversight of data science health research across national borders. They should rigorously engage a broad range of stakeholders including those whose voices are typically drowned out in global discourse. Innovations in virtual meeting technologies should enable cost-effective, frequent, and sustained global engagement of stakeholders.

### Reduce digital inequity and increase volume and diversity of African datasets

Data science relies on large repositories of data generated by individuals as they engage with the healthcare system, during activities of daily living, and participation in research^71^. Healthcare data may derive from electronic health records, surveillance data, diseases’ registries, etc., while other datasets may be derived from wearable devices and other digital footprints. Large-scale genomic, transcriptomic, proteomic, and other omics research projects are also generating huge amounts of data for data science health research. Despite interventions like H3Africa and DS-I Africa, more of these data are still being generated in HIC compared to LMIC^27,72^. The resulting digital data inequity is pervasive and growing worse^73^. Digital health innovations also contribute to the widening inequities because of the “inverse care law” which postulates that well-resourced individuals are more likely to be aware of and utilize digital health interventions^74^. Unaddressed, these inequities will lead to severe and adverse health outcomes for majority of the world’s population^75^. Urgent, sustained, large-scale efforts are required to reverse this trend for the sake of equity and justice.

Multi-level interventions guided by frameworks for digital health equity such as the digital determinants of health (DDOH) would be useful for identifying the barriers and facilitators, and guide meaningful interventions to increase the volume of digital health data generated in Africa^73^. General investments in healthcare systems, implementation of electronic health records, improvements in diseases’ registries, and broader utilization of digital systems will increase the amount of digital data generated by African populations. Additional systematic interventions that are similar to but substantially larger than programs like H3Africa and DS-I Africa are needed to ensure that African countries, at a minimum, keep up with the high volume of omics and other data types being generated in HIC for data science.

### Reduce and eliminate algorithmic bias, data colonization, and extractive research

Data science technologies produce algorithmic bias by replicating and reinforcing societal biases that benefit or disadvantage certain individuals or groups. This results in structural, racial and ethnic biases in the HIC where most data science technologies are developed^76^. These algorithmic biases  coupled with the lack of equity and diversity in the foundational datasets used to develop, train, and validate data science algorithms lead to algorithmic deprivation, discrimination, and distortion^77–81^. Other concerns, particularly with respect to data science in Africa, are data colonization and extractive research^82^. Biased and inequitable algorithms lead to ethically, socially, politically, and economically undesirable outcomes in health research and health care, and can negatively affect perceptions of fairness, acceptability, and trust in applications derived from data science health research. These have the potential of denying populations that are most in need, the benefits of data science health research^76^. These harms are unpredictable and may not be remediable post hoc, they therefore require vigorous and robust attention a priori^51,52,76^.

Many approaches have been recommended to reduce or eliminate algorithmic bias in data science health research^79,81,83–85^. These include improving the diversity of data scientists through targeted capacity building programs, creation and implementation of guidelines and policies, implementation of programs to detect and rectify algorithmic bias, training data scientists on health research ethics^52,85–87^. These interventions require long-term commitments that go beyond the typical duration of many HIC research grant award mechanisms. They also require strong commitment by national governments, local institutions, and research sponsors. Novel strategies for supporting the development of personnel, resources, and infrastructure for data science health research in Africa that are aligned with clear goals and objectives, rather than just utilizing frameworks developed and used in the substantially different health research ecosystems of HIC are desperately needed.

Given the scope of data science health research, its potential to improve health outcomes, engender more equitable research participation, reduce marginalization, and utilize heterogeneous data types, all stakeholders must be urgently engaged in development of the most efficacious governance frameworks for it.
